# Reports on Hospitals of the United Kingdom

**Published:** 1914-01-31

**Authors:** Henry Burdett


### REPORTS ON

### Hospitals of the United Kingdom.

By SIR HENRY BURDETT, K.C.B., K.C.V.O.

SERIES III.

### ADDENBROOKE'S HOSPITAL, CAMBRIDGE.

This hospital was established in 1719. The first buildings were erected in 1740, and it was constituted a general hospital in 1766 by Act of Parliament, in which year the hospital was first opened for the reception of patients. Just prior to 1820 new wings were added through the generosity of a Cambridge bookbinder, John Bowtell, who left a legacy to the hospital. In 1864-65 the hospital was entirely reconstructed and enlarged from plans of Sir M. Digby Wyatt and Sir G. M. Humphry, the details of which will be found on page 269 of The Hospital, July 16, 1892. This plan had the merit of securing that the best work was put into the building and equipment of the new hospital, though the plans were naturally faulty when judged by the requirements of a modern hospital. In our report we called attention to the condition of the floors of certain wards, which have been relaid and are at present satisfactory. Since that date many modifications and readjustments have taken place, including new quarters for the matron. In 1878 further wards were added, and in more recent years the first Nurses' Home was erected, which, since the year when a larger and up-to-date home was built, has been occupied by the domestic staff.

### A Practical Vice-Chairman.

At the present time extensive alterations and additions to the buildings are in course of erection. These include a clinical laboratory erected in memory of the late secretary, Mr. John Bonnett, which will shortly be ready for occupation. This laboratory was planned by Messrs. Everard, Son and Pick, architects, of Leicester, who have also been entrusted with the planning of the new out-patients' department, the wards for children and an addition to the existing Nurses' Home, the new boiler-house with new boilers, and a central electric lift.

The hospital is fortunate in having for Vice-Chairman the Rev. J. B. Lock, who has a practical knowledge of buildings and plans which cannot fail to be of immense advantage to the hospital. When the various buildings and improvements in course of erection are completed and occupied, Addenbrooke's Hospital should possess many of the requisites of a modern up-to-date hospital and be made worthy of the University of Cambridge.

### The Semi-Convalescent Home.

A good feature of this hospital is its Home of Recovery, or semi-convalescent home, opened at Hunstanton in 1899. It is maintained entirely for the reception of patients from the hospital, eighty-nine of whom were admitted in 1912. There can be no doubt that an actively administered and up-to-date semi-convalescent home where patients requiring dressings can be sent from the wards to the best air outside at the earliest possible moment hastens recovery and adds materially to the earning power of the majority of such patients. Seeing that the number of in-patients last year amounted to 1,961, and that only eighty-nine of them were admitted to the Home of Recovery, we conclude that further accommodation at Hunstanton or elsewhere is called for.

### Some Fruits of Good Management.

Great improvements have taken place in the economical administration of the hospital in recent years owing to the adoption by the committee of a system of control introduced by the Secretary-Superintendent, Mr. Richard J. Cole. This system has taken some time to get into smooth working, but now that this is accomplished it has proved of great advantage, and has, we understand, rendered good service in many directions. The arrangement of the stores and the organisation of the store-rooms and other departments are good. We found the quality of the nursing well up to the high standard which has prevailed at this hospital since the days of the lamented Miss Fisher, who first established a training school for nurses at Addenbrooke's in 1877.

### A Hint on Planning.

In planning the sanitary annexes to the new blocks on the third floor, and elsewhere where reconstruction will take place, the details should provide for the placing of the soil and mackintosh sinks and w.c. adjuncts all together, so that they may be quite apart and separate from the bathroom and lavatory accommodation.

### The Medical School.

Under this head the report for 1912 contains a paragraph stating that this hospital has from very early times done good service in training medical men. It proceeds: "By gradual growth there is now a large and important medical school in connection with the hospital. . . The hospital is recognised as a place of clinical study by the University and by the various examining bodies in England and elsewhere." Since 1900 the Regius Professor of Physic and the Professor of Surgery have been elected as additional members of the medical staff. It is essential, if Addenbrooke's Hospital is adequately to fulfil the claims upon it and to live up to the standard of medical efficiency set forth in the report, that the honorary medical staff shall consist of physicians and surgeons of the highest standing and accomplishments. Cambridge cannot have a hospital up to date as a clinical centre or as a place containing adequate clinical material for the instruction, or even for the examination of advanced students, unless the medical and surgical officers are efficient as a teaching staff of the highest order. We mention this point because when the whole of the work now in hand is completed and the hospital contains the necessary facilities required to fulfil the claims we have quoted from the report, the standard of qualification for members of the staff must be equal at least to that enforced at other important educational centres.

### A Welcome Change.

This hospital has undergone many reconstructions and changes in thirty years. It has survived the dangerous period when for some twenty-five years or more its destinies were in the hands of a committee which was so conservative as to keep the hospital back in nearly every department of its work. That period has now happily terminated. Those responsible for the administration of Addenbrooke's Hospital at the present time have shown a progressive spirit well calculated to maintain the highest efficiency and to increase the reputation of this ancient hospital. In the course of our visit, on October 25, 1913, we had an opportunity of realising the thoroughness with which the plans for the new sections have been gone into, and we found that the Vice-Chairman, at any rate, possesses the rare advantage of understanding plans thoroughly: Mr. Lock, in fact, realises exactly what plans mean. He is quick to recognise suggestions that are improvements and to negative others that have either been provided for or are immaterial. All this is very important, especially now that the whole trend of the administration at Cambridge to-day is progressive from top to bottom.

### The Many Improvements Demonstrated.

We congratulate the committee and all connected with the administration upon the marked improvement nearly everywhere apparent. Those who doubt this fact should take "A Report on Certain Proposed Changes in the Constitutional, Financial, Nursing, Administrative, and Structural Arrangements of Addenbrooke's Hospital, Cambridge," dated June 1898, and check off as we have done how far and to what extent the recommendations that report contains have been adopted and carried out. Such a course must convince doubters and tend to bring a due measure of praise and recognition to all who have helped, or are helping, to make Addenbrooke's Hospital what it ought to be.

---

**The Bedford County Hospital will be reported on next week.**

